# Mapping evidence on malnutrition screening tools for children under 5 years in sub-Saharan Africa: a scoping review protocol

**DOI:** 10.1186/s13643-020-01309-6

**Published:** 2020-03-09

**Authors:** Tlharihani Phisac Maphosa, Delarise Maud Mulqueeny, Ernest Osei, Desmond Kuupiel, Tivani P. Mashamba-Thompson

**Affiliations:** 1 Discipline of Public Health Medicine, School of Nursing and Public Health, University of KwaZulu-Natal, Howard College Campus, 2nd Floor of George Campbell Building, Durban, 4001 South Africa; 2 Department of Social Science, Gender and Education, School of Education, University of KwaZulu-Natal, Room 01-032, 121 Marianhill Rd, Pinetown, Durban, 3605 South Africa; 3 Research for Sustainable Development Consult, Sunyani, Ghana; 4 Department of Public Health, University of Limpopo, Polokwane, Limpopo Province South Africa

**Keywords:** Malnutrition screening tools, Nutritional screening tools, Children under 5 years, Sub-Saharan countries

## Abstract

**Background:**

In sub-Saharan Africa (SSA), malnutrition remains a major public health challenge, particularly among children under 5 years of age. Despite nutritional screening tools being developed and available to detect early malnutrition in under five-year-old children, malnutrition continues to be a health concern. However, the level of evidence on nutritional screening tools for predicting early malnutrition at the community level in a high disease burden setting is unclear. The objective of this scoping review is to systematically map the evidence on malnutrition screening tools for children under 5 years in sub-Saharan Africa (SSA) and to identify knowledge gaps.

**Methods:**

The proposed study will be guided by an improved Arksey and O’Malley’s framework, Levac *et al*. 2010 recommendations, and the 2015 Joanna Briggs Institute guidelines. We will conduct a systematic search of relevant imperial sources of evidence from the following databases: CINAHL with full text, Academic search complete via EBSCOhost, Google Scholar, Science Direct, and PubMed. We will search for grey literature from the following humanitarian and aid organization websites: World Health Organization (WHO), The United Nations International Children’s Emergency Fund (UNICEF), and governmental departments. Following the database searches and title screening, eligible sources of evidence will be exported to an EndNote X9 reference library. Thereafter, duplicate articles will be removed in preparation for abstract and full article screenings. Data from the included sources of evidence will be extracted, and the emerging themes will be analyzed. The relationship between the emerging themes and the research questions will be critically examined. The quality of the included sources of evidence will be determined by using the Mixed Method Appraisal Tool (MMAT) version 2018. The search results will be presented in adapted Preferred Reporting Items for Systematic Reviews and Meta-Analysis: Extension for Scoping Reviews chart (PRISMA-ScR).

**Discussion:**

We anticipate finding relevant literature on malnutrition screening tools for children under 5 years in SSA. This study is likely to reveal research gaps, which could guide future research on malnutrition screening tools.

## Background

Malnutrition remains a major public health challenge, particularly among children under 5 years of age [1]. Globally, about 17 million children under 5 years of age suffer from severe acute malnutrition (SAM) and the majority live in southern Asia and sub-Saharan Africa (SSA) [2]. In this scoping review, SSA refers to 46 African countries that are fully or partially located in the south of the Sahara [3]. The United Nations International Children’s Emergency Fund (UNICEF), the World Health Organization (WHO), and the World Bank estimate global and regional child malnutrition reports that we are still far from a world free of malnutrition among children under-five, that there is insufficient progress in achieving the World Health Assembly targets set for 2025 and the 2030 Sustainable Development Goals [4]. As children living in SSA are at a higher risk of malnutrition, improved screening, availability of appropriate paediatric screening tools and their correct use, check-ups, and timely interventions to improve the health outcomes of children is pivotal [5, 6].

Malnutrition screening tools (MST) are instruments used for early detection of patients who are nutritionally at risk and those who are already malnourished [7]. Since 1995, several MST for early detection of malnutrition in hospitalized children have been developed and proposed for use (high-income countries (HIC) [8, 9]). In SSA, MST commonly used to classify malnutrition indicators (wasting, stunting, and underweight) in children under 5 years of age include the use of anthropometric assessments such as height-for-age (HFA), weight-for-height (WFH), weight-for-age (WFA), and mid-upper arm circumference (MUAC) [10, 11]. Reasons due to the inadequate or incorrect use of malnutrition screening tools include lack of training, health professional’s insufficient awareness, misunderstanding of available tools, shortages of equipment or personnel, and lack of nutritional information given to caregivers. It is important that MST follow effective and efficient processes, as the correct interpretation and use of these tools are crucial to improving early detection and care linkage in malnourished children [12, 13].

An initial scoping search of the literature to determine whether systematic reviews and recommendations on our research questions that have been published has found these manuscripts more in HIC. Suitable screening tools for children are scarce with no consensus on the best method to assess their risk of malnutrition despite several recommendations on the importance of its early identification [14–16]. Moreover, due to a lack of simple validated methods, malnutrition screening is not widely and correctly performed [17, 18]. Research studies conducted in South Africa and Uganda have shown that health professionals have inadequate knowledge of available nutritional status interpretation tools as gaps have been identified [19, 20]. Moreover, the level of evidence on nutritional screening tools to predict early malnutrition at community levels in high disease burdened settings such as SSA is unclear.

Hence, as this is a broad topic, a scoping review was found to be most useful over a systematic review to map a range of literature that exists and would aid in focusing the research questions by charting existing research findings and identifying research gaps [21, 22]. It is anticipated that the results of this review will reveal gaps to guide future research and inform policymakers to ensure the successful implementation of current and future MST for children under-five in disease burdened settings. The results will also ensure that health professionals and educators are aware of the MST that need to be included in local medical curricula. In this sense, there will be early identification of children who are at risk of malnutrition, and it is essential because it allows appropriate nutritional interventions to prevent malnutrition and its consequences.

## Objective

The objective of this scoping review is to systematically map the evidence on malnutrition screening tools for children under the age of five in sub-Saharan Africa (SSA) and to identify knowledge gaps.

## Methodology

### Design

A scoping review method was selected as it outlines different types of evidence in the area of interest and highlights gaps for further research. Based on this, the current scoping review uses an enhanced Arksey and O’Malley’s framework, Levac *et al.* 2010 recommendations, and the 2015 Joanna Briggs Institute guidelines to guide the methodology of this scoping review [23–25]. The framework involves (1) identifying the research question, (2) identifying relevant sources of evidence, (3) selection of sources of evidence and eligibility, (4) charting the data, and (5) collating, summarizing, and reporting the results. The results of this proposed study will be presented according to the Preferred Reporting Items for Systematic Reviews and Meta-Analysis: Extension for Scoping Review guidelines (PRISMA-ScR) [26]. This protocol has not been registered a priori. The PRISMA-P checklist was used for this protocol (Additional file 1) [28, 29].

#### Identifying the research question

The research questions are based on the research objective that was formulated using the Population-Concept-Context (PCC) framework designed by the Joanna Briggs Institute [25]. It was used to determine the eligibility of the scoping review question as shown in Table 1.

**Table 1:** Eligibility criteria

Criteria	Determinants	Inclusion criteria	Exclusion criteria
**Population**	**Under-five** refers to children who are less than 5 years old.	Articles presenting evidence on children under 5 years.	Articles presenting evidence on children with developmental delays (e.g., children with cerebral palsy).
**Concept**	**Malnutrition screening tools** refer to tools used to screen patients who are at risk of malnutrition [7]. These tools include height-for-age (HFA), weight-for-height (WFH), weight-for-age (WFA), and mid-upper arm circumference (MUAC).	Articles reporting evidence on malnutrition screening tools for children under 5 years. Articles published between 2010 and 2019 will be considered in order to obtain the most recent information on our research topic	Articles and studies that did not include specificity on malnutrition screening tools.
**Context**	**Sub-Saharan Africa** refers to 46 African countries that are fully or partially located south of the Sahara [3].	Articles reporting evidence from SSA.	
**Sources of evidence**		The review will include empirical literature and grey literature presenting evidence on malnutrition screening tools for children under 5 years in SSA.	

The primary research question is what evidence exists on malnutrition screening tools for children under 5 years living in sub-Saharan Africa?

The secondary research questions are:

- What malnutrition screening tools are used for children under 5 years in sub-Saharan Africa?
- What evidence exists regarding the performance of malnutrition screening tools for children under 5 years in sub-Saharan Africa?

#### Identifying relevant sources of evidence

The review will include empirical literature and grey literature that present evidence on malnutrition screening tools for children under 5 years in SSA. We will search for relevant empirical literature from the following electronic databases: CINAHL with full text, Academic search complete via EBSCOhost, Google Scholar, Science Direct, and PubMed. To improve the quality and reduce errors of the electronic search, the search strategies were peer reviewed using the PRESS 2015 Evidence-Based Checklist [30]. The keywords informing the searches are malnutrition screening tools, nutritional screening tools, children under 5 years, and sub-Saharan countries. We will also search for grey literature from the following sources: humanitarian and aid organization websites such as The World Health Organisation (WHO), The United Nations International Children’s Emergency Fund (UNICEF), and governmental departments’ websites. These websites will be searched for current policies, guidelines, statistics, and interventions. The reference lists of the included articles will be thoroughly searched for relevant articles by a research assistant. Additionally, when relevant sources of evidence are inaccessible, authors will be contacted for the actual articles. A University of KwaZulu-Natal (UKZN) librarian specializing in developing searches in the health sciences was consulted to assist in the development of this search strategy. We have piloted the search strategies to check for the appropriateness of the selected databases and keywords using the Boolean terms “AND” and “OR” to separate the search terms. The results thereof are found in Table 2.

**Table 2:** Draft search for PubMed/MEDLINE

Search engine used	Keywords search
PubMed	((“malnutrition”[MeSH Terms] OR “malnutrition”[All Fields]) OR ((“diagnosis”[Subheading] OR “diagnosis”[All Fields] OR “screening”[All Fields] OR “mass screening”[MeSH Terms] OR (“mass”[All Fields] AND “screening”[All Fields]) OR “mass screening”[All Fields] OR “screening”[All Fields] OR “early detection of cancer”[MeSH Terms] OR (“early”[All Fields] AND “detection”[All Fields] AND “cancer”[All Fields]) OR “early detection of cancer”[All Fields]) AND tools[All Fields])) AND ((“child”[MeSH Terms] OR “child”[All Fields] OR “children”[All Fields]) AND under[All Fields] AND five[All Fields]) AND (“africa south of the sahara”[MeSH Terms] OR (“africa”[All Fields] AND “south”[All Fields] AND “sahara”[All Fields]) OR “africa south of the sahara”[All Fields] OR (“sub”[All Fields] AND “saharan”[All Fields] AND “africa”[All Fields]) OR “sub saharan africa”[All Fields])

#### Selection of sources of evidence and eligibility

**Fig. 1:**
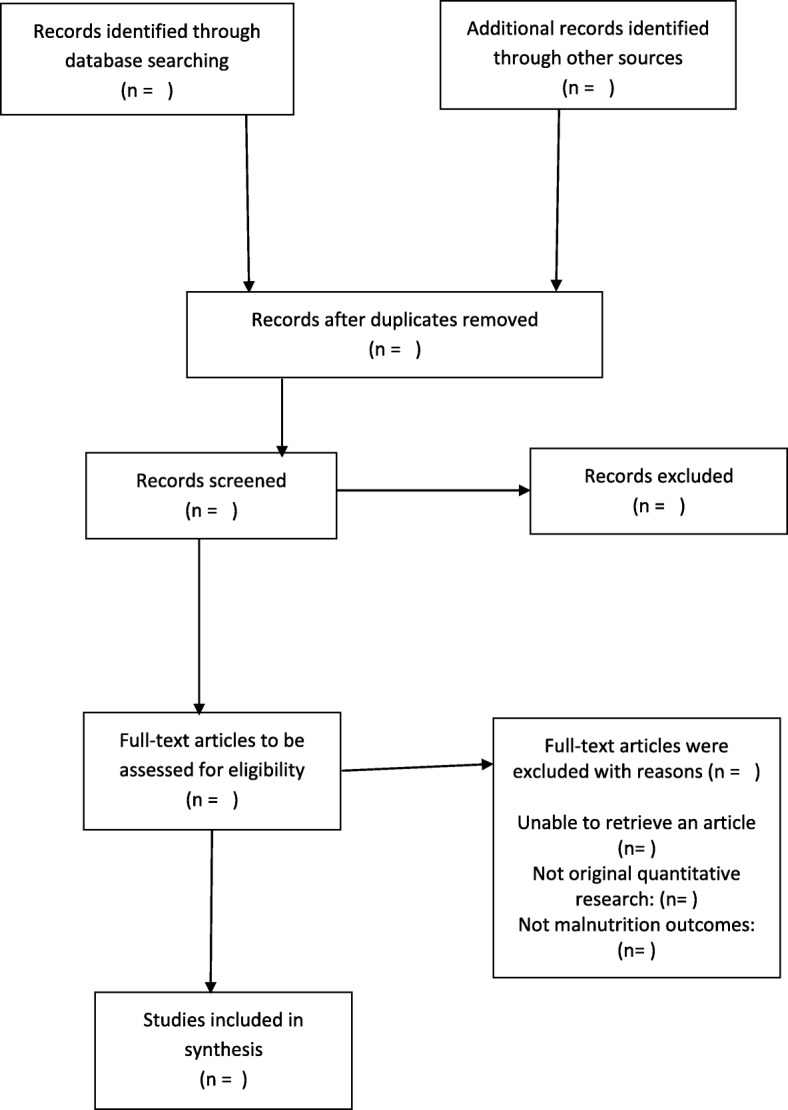
Selection of sources of evidence. PRISMA ScR flowchart which demonstrates the literature search and Selection of Sources of Evidence

To fine-tune the selection of the sources of evidence process and to improve consistency, a pilot test will be conducted prior to the review process commencing. There will be no language restrictions applied to the literature search. About 60 publications will be used for this procedure. To reduce any selection bias, screening of study titles and abstracts from the databases listed above will be conducted by two investigators (TPM and EO) independently. The relevant sources of evidence will be identified with the guidance of the inclusion and exclusion criteria. The eligible sources of evidence will then be exported to an Endnote X9 library created specifically for this review. All duplicates identified will be deleted before sharing the Endnote library with the two reviewers. An abstract screening form with questions will be developed based on our eligibility criteria. Discrepancies between reviewers at the title and abstract stage will be resolved through discussions until a consensus is reached. Following the title and abstract screening, full articles will be screened by the two reviewers (DMM and TPM) independently in parallel. Discrepancies between reviewers at the full article stage will be resolved by a third screener (DK). To ensure the reproducibility of the study, the references of excluded sources of evidence and the rationale for exclusion will be provided in an additional file of the completed review. Figure 1 presents an example of our planned selection of sources of evidence [26, 27].

### Eligibility criteria

To ensure that relevant sources of evidence are selected for this review, the study selection process will be guided by the eligibility criteria as specified under the inclusion/exclusion criteria. The sources of evidence will include information from empirical literature and grey literature that present evidence on malnutrition screening tools for children under 5 years in SSA.

1. ***Population***

- We will include articles presenting evidence of children under 5 years. Under-five in this study refers to children who are less than 5 years old.
- We will exclude articles presenting evidence on children who are under-five but with developmental delays (e.g., children with cerebral palsy). This is because the tools that are used to screen children at risk of malnutrition are different from the ones used in children without delays.

2. ***Concept***

- Articles reporting evidence on malnutrition screening tools will be included in this study. Malnutrition screening tools refer to tools used to screen patients who are at risk of malnutrition [7].
- Articles and studies that did not include the specificity of malnutrition screening tools will be excluded.
- Articles published between 2010 and 2019 will be considered in order to obtain the most recent information on our research topic.

3. ***Context***

- We will include articles reporting evidence from SSA. Sub-Saharan Africa refers to 46 African countries that are fully or partially located south of the Sahara [3].

#### Charting/extraction of data

A data charting form will be created using a Google form where all the necessary extracted data from the included sources of evidence can be populated (Table 3). The standard bibliographical information (i.e., authors, title, and year of publication), geographical setting, study setting, study design, and aim of the study will be reported in the form. For each of the included sources of evidence, information on the target population, type of intervention, nature of the outcome, key findings, most significant findings, conclusions, and notes will also be tabled. The data extraction will be conducted by two investigators (TPM and EO) independently, and the extraction form will continually be updated to ensure accuracy and consistency of extracted data. All disagreements between investigators in the data extraction process will be addressed through discussion until consensus is reached. Persistent disagreements will be resolved by involving a third screener (DK).

**Table 3:** Data extraction table

Items	Description of items	Page No./column
Author and date		
Country		
Aim of the study		
Geographical setting		
Study design		
Study setting		
Study population		
Duration of the study		
Type of intervention		
Nature of the outcome		
Key findings of the study		
Most significant findings of the study		
Conclusions		
Notes		
Issues for contacting authors		

#### Collating, summarizing, and reporting the results

We will present a narrative account of the findings from the included sources of evidence and present themes. Thematic content analysis will be employed to extract the themes, which will be critically examined in relation to the study research question, the aim of the study, literature, gaps for future research, and MST for under 5 years in SSA countries. The implications of the study results for future research, policy, and practice will be examined and reported on.

#### Quality appraisal

We will utilize the Mixed Method Appraisal Tool (MMAT) version 2018 to appraise the methodological quality of included sources of evidence [31]. The criteria for the appropriateness of included sources of evidence will be determined by the aim of the study, appropriate methodology, study design, recruitment strategy, and appropriate sampling technique. Other items include suitable data collection procedures, appropriate data analysis, appropriate data interpretation, presentation of findings, discussion, and conclusions of the author from the included articles. A quality appraisal will be carried out to examine the strengths, weaknesses, and quality of research evidence and presented for each included article. The quality of all the included articles will be calculated and rated using the MMAT guidelines with 25% accounting for low-quality articles, 50% being average, 75% being above average, and 100% being high average. This will ensure that the study designs of the included sources of evidence are appropriate for the research objectives. The quality assessment will also assist us in reporting on the risk of bias and the quality of evidence of the included sources.

#### Differences between the protocol and the review

All differences between the protocol and the final research study will be reported together with the rationale for these changes. The consequences of these modifications on the magnitude, direction, and validity of the outcomes will also be presented [32].

## Discussion

Timely treatment of malnutrition in children of five years of age at primary healthcare facilities could prevent 500,000 deaths annually [33]. Hence, the importance of best practices regarding malnutrition screening tools for children under 5 years. This study will encourage the correct use of MST for children under-five and its findings could assist with the achievement of the Sustainable Development Goals (SDGs). As the focus of this review is on malnutrition screening tools, sources of evidence that do not focus on malnutrition screening tools will be excluded since such data is irrelevant and will not address the study research questions.

The limitations of this research study include the following: (1) it may omit sources of evidence that include participants older than five and may result in the exclusion of important sources of evidence and (2) including sources of evidence published between 2010 and 2019 could introduce the risk of publication bias. However, this period was chosen because it will represent the most recent information on our research topic. The study will focus on children under the age of 5 years in SSA as literature has shown that this region has the highest under-five mortality rate in the world [34]. Moreover, prior to 2011, a Road to Health Card (RTHC) was used as an essential monitoring tool for children under five-year-old’s health. However, the Road to Health Booklet (RTHB) is currently being used to monitor children under-five health and its correct use assists in the early detection of malnutrition [35]. Hence, the limitation of our literature to 2010 and 2019 will result in us obtaining the most recent information during this period.

The current scoping review strength is that it addresses objectives that are important for patients, clinicians, and policymakers. We expect that the scoping review results will provide a comprehensive overview of the evidence on the topic and highlight areas where evidence is controversial or missing. Additionally, it will provide key information to policymakers and health professionals interested in planning, funding, and delivering evidence-based effective interventions aimed at preventing malnutrition in children under 5 years. Moreover, identified research gaps could inform future studies and guide policy decisions to enhance healthcare outcomes in SSA. We plan to disseminate the study’s findings in peer-reviewed journals and at conference proceedings that focus on nutritional and disease screening. Additionally, the study findings will be disseminated to professionals and stakeholders involved in malnutrition prevention and treatment.

## Supplementary information

**Additional file 1.** PRISMA-P (Preferred Reporting Items for Systematic Review and Meta-Analysis Protocols) 2015 checklist: recommended items to address in a systematic review protocol*.

## Data Availability

All data generated or analyzed during this study will be included in the published scoping review article and will be available upon request.

## Abbreviations

HFA: Height-for-age
HIC: Higher income countries
MST: Malnutrition screening tools
MUAC: Mid-upper arm circumference
PCC: Population-Concept-Context
PRISMA ScR: Preferred Reporting Items for Systematic Reviews and Meta-Analysis: Extension for Scoping Reviews
SSA: Sub-Saharan Africa
UNICEF: United Nations International Children’s Emergency Fund
WFA: Weight-for-age
WFH: Weight-for-height
WHO: World Health Organization
